# Chronic Liver Disease and the Risk of Osteoporotic Fractures: A Meta-Analysis

**DOI:** 10.7759/cureus.10483

**Published:** 2020-09-16

**Authors:** Diego F Hidalgo, Boonphiphop Boonpheng, Sehrish Sikandar, Lubna Nasr, Jessica Hidalgo

**Affiliations:** 1 Geriatrics, Jackson Memorial Hospital, University of Miami, Miami, USA; 2 Internal Medicine, East Tennessee State University, Johnson City, USA; 3 Geriatrics, Miami Geriatric Research Education and Clinical Center Veterans Successful Aging for Frail Elders (VSAFE), Miami, USA; 4 Geriatrics, University of Miami Miller School of Medicine, Miami, USA; 5 Internal Medicine, San Francisco de Quito University, Quito, ECU

**Keywords:** chronic liver disease, osteoporotic fractures, osteoporosis

## Abstract

Introduction

Chronic liver disease (CLD) causes more than 1 million deaths every year and remains a pandemic in the last decade affecting more than 600,000 patients in the United States. Previous studies found patients with CLD had increased risk of osteoporosis, so fractures were inferred to be complications of this condition. The aim of this meta-analysis is to summarize the best evidence that correlates CLD patients and the risk to develop osteoporotic fractures versus control patients without CLD.

Methods

A review of the literature using MEDLINE and EMBASE database was performed during December 2017. We included cross-sectional and cohort studies that reported relative risks (RR), odds ratios (OR) and hazard ratios (HR) comparing the risk of developing osteoporotic fractures among patients with CLD versus patients without CLD. Pooled OR and 95% confidence interval (CI) were calculated using generic inverse- variance method. The Newcastle-Ottawa scale was used to determine the quality of the studies. Effect estimates from the individual study were extracted and combined using the random-effect, generic inverse variance method of DerSimonian and Laird.

Results

After the review of the literature, seven studies fulfilled the eligibility criteria established during the analysis. Significant association was found between CLD and osteoporotic fractures with a pooled OR of 2.13 (95% CI, 1.79 - 2.52). High heterogeneity among the studies was found (I2=88.5). No publication bias was found using Egger regression test (p=0.44).

Conclusion

We found a significant association between CLD and the risk of developing osteoporotic fractures. The calculated risk was 2.13 times higher for patients with CLD when compared with controls. The results showed high heterogeneity but no publication bias. More prospective studies are needed to fully understand the mechanisms involved in loss of bone density and osteoporotic fractures in order to improve the morbidity associated with this disease.

## Introduction

Chronic liver disease (CLD) is a progressive deterioration of liver function. It is shown through a process of worsening fibrosis and formation of regeneration nodules over a period of months. Initially the fibrosis may be reversible but, if not treated, it can lead to irreversible fibrosis, regeneration nodules formation and hence the development of cirrhosis [1]. The trend of chronic liver disease in the US is changing swiftly. Currently, it is the fourth leading cause of death in the US among adults 45 to 64 years old. According to the National Vital Statistics Report of 2017 from the Center for Disease Control and Prevention in the United States, approximately 4.5 million adults suffered from chronic liver disease and cirrhosis; which represents 1.8% of the adult population [2]. The rate of mortality from chronic liver disease and cirrhosis was 12.8 deaths per 100,000 population, equaling about 41,473 deaths in number [3].

The most common risk factors for chronic liver disease include excessive alcohol consumption, hepatitis B and C, obesity, diabetes mellitus, and metabolic syndrome [4]. Since the liver is an organ involved in various mechanisms of metabolism, chronic liver disease can lead to secondary osteoporosis which affects about 30% of patients suffering from this disease [5,6]. A number of factors are responsible for osteoporosis, including alteration in the metabolism of vitamin D and calcium, vitamin K deficiency, hormonal dysregulation, release of cytokines and deficiency of insulin-like growth factor 1 (IGF-1) [7]. Dysregulation of these processes may lead to disorders in bone homeostasis which can ultimately lead to osteopenia, osteoporosis and hence causing osteoporotic fractures [8].

Patients with confirmed chronic liver disease should be screened for osteoporosis as they are considered medium or high risk based of different factors. Serum vitamin D levels can also be obtained in order to correct the modifiable risk factors like calcium and vitamin D deficiency, smoking, alcohol abuse, and malnutrition [9]. The quality of the trabecular bone at the lumbar spine and hip can be obtained by densitometry tests. However, ascites can affect the densitometric accuracy of the tests by causing a fluid artifact that can falsely lower the bone mineral density measurements [10,11]. Many patients with osteoporosis go undiagnosed in the primary care clinic, mainly due to the lack of diagnostic tools. Multisite bone ultrasound methods are new tools that can potentially be used successfully in the future to diagnose low bone density, which can be more easily available in primary care settings [12].

The aim of this meta-analysis is to summarize the best evidence that correlates CLD patients and the risk of developing osteoporotic fractures versus control patients without CLD.

## Materials and methods

Search strategy

A review of the literature using MEDLINE and EMBASE database was performed during December 2017 by two investigators (DH and BB). The search strategy included terms and synonyms for “CLD,” “osteoporosis,” and “fractures.”

This study meets the criteria checklist in accordance with the Preferred Reporting Items for Systematic Reviews and Meta-Analyses (PRISMA) statements.

Selection criteria

Any study, in order to be selected for this meta-analysis, had to fulfill the following parameters:

-Cross-sectional and cohort studies published by the two major databases used related to patients with CLD caused by cirrhosis, primary biliary cholangitis, and primary biliary cirrhosis. Subjects without CLD were used as comparators in cohort and cross-sectional study. 

-Relative risks (RR), odds ratios (OR), and hazard ratios (HR) comparing the risk of developing osteoporotic fractures among patients with CLD versus patients without CLD. 

-Pooled OR and 95% confidence interval (CI) were calculated using generic inverse-variance method. 

**Figure 1:**
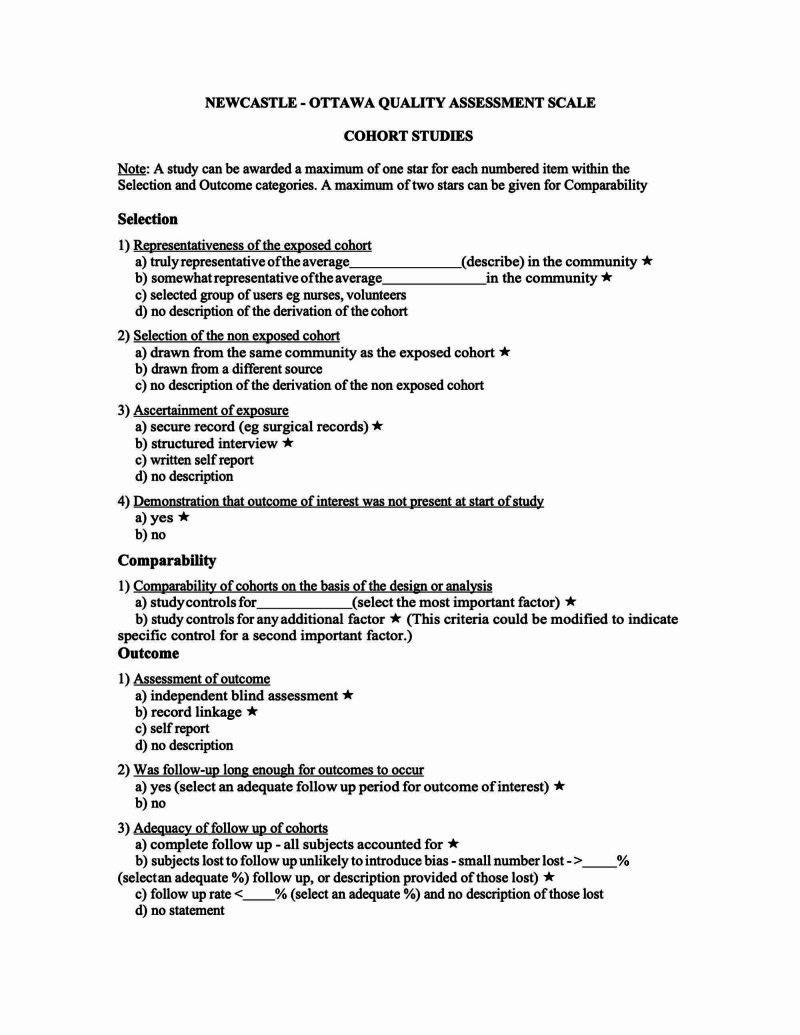
Newcastle-Ottawa quality assessment scale Adapted from [16].

The Newcastle-Ottawa scale (Figure 1) was used by the investigators independently to determine the quality of each study. This scale evaluates each study in terms of participants selection (minimum score is 0; maximum is 4), comparability (minimum score is 0; maximum is 2), and the ascertainment of the exposure of interest for case-control studies, and the outcome of interest for cohort studies (minimum score is 0; maximum is 3) [13,14,15]. Newcastle-Ottawa scale contains eight items within three domains and the total maximum score is 9. A study with a score from 7-9 has high quality, 4-6, high risk, and 0-3 very high risk of bias [16]. Also, the effect estimates from the individual study were extracted and combined using the random-effect, generic inverse variance method of DerSimonian and Laird [17].

Data extraction

The data collection used in this study was through the use of Microsoft Excel (Microsoft Corporation, Redmond, Washington, US) summarizing the most relevant information obtained from these studies. The characteristics included in these studies contained the first author's last name, country, study design, year of publication, demographics, total number of participants, characteristics of the participants, the method used to diagnose CLD, the method used to determine fractures, adjusted effect estimates with 95% CI, confounder adjustment and the Newcastle-Ottawa quality assessment scale (Table 1).

**Table 1:** Characteristics of studies included PBC: Primary Biliary Cirrhosis, CP: Chronic Pancreatitis, HE: Hepatic Encephalopathy, LC: Liver Cirrhosis, ICD: International Classification of Diseases, HR: Hazard Ratio, OR: Odds Ratio, IRR, CI: Confidence Interval, GPRD: General Practice Research Database.

Table 1: Characteristics of studies included							
	Chen et al. [18]	Solaymani et al. [19]	Tsai et al. [20]	Bang et al. [21]	Boulton et al. [22]	Fan et al. [23]	West et al. [24]
Country	Taiwan	United Kingdom	Taiwan	Denmark	United Kingdom	China	United Kingdom
Study design	Longitudianl Retrospective Cohort study	Population-Based Cohort Study	Nationwide Population- Based Study	Retrospective Cohort Study	Population Based Cohort Study	Meta analysis	Population Based Cohort Study
Year	2017	2006	2012	2014	2004	2017	2010
Number of participants	692,231	10,132	3764	Total: 228459; 20,769 Patients with cirrhosis, and 207,690 controls	A total of 201 participants with 85 PBC patients and 116 controls	1643 PBC patients and 10921 controls	A total of 4787 subjects with cirrhosis and 46,789 appropriately matched controls
Participants	The investigation included two studies. Study I: 3941 adults patients aged ≥20 years in 2000–2003 with primary diagnosis of cirrhosis of the liver and at least two visits for medical care frequency-matching procedure (by age and sex) with no previous medical records of LC. Study II: 688290 hospitalised patients with fracture in 2004–2013 with a history of LC within 24 months pre-fracture. Thirty-day in-hospital mortality, septicaemia, and acute renal failure after fracture were considered post-fracture outcomes and were compared in patients with fracture with and without LC in the nested fracture cohort study.	930 adults patients aged ≥20 years with Primary Biliary Cirrhosis and 9202 controls based in The General Practice Research Database (GPRD) between June 1987 and April 2002	4962 HE with cirrhosis patients over the 10-year study period. After excluding patients diagnosed at <20 years of age (n = 131) or who had a fracture before enrollment (n = 1067), the sample consisted of 3764 patients for analysis. Patient age ranged from 20 to 100 years, with a median age of 53years.	Patients diagnosed with cirrhosis or CP were identified from the Danish National Patient Register. Each patient was matched to 10 age- and sex-matched controls using the Danish Civil Registration System. For both the cases and controls, an event was defined as any fracture that happened in the period from January 1, 1995, to December 31, 2010.	Patients attending Nottingham University and City Hospitals with PBC were included. Patients who had had a liver biopsy reported as showing PBC or who had had a raised antimitochondrial immunoglobulin G (IgG) antibody titre of greater than 1:50 on a sample requested by a gastroenterologist were identified from a computerized pathology database from 1991 to 1999 and immunology database from 1993 to 1999 respectively. A control group of patients was identified from the control series of a large population-based case–control study of myocardial infarction in women previously conducted in the same geographical area.	Specific for each article. Does not mention any specific number either per article or total.	Patients with a diagnostic of therapeutic code for cirrhosis, esophageal varices and/or portal hypertension within the General Practice Research Database=GPRD between June 1987 and April 2002 and records of up to 10 age-, sex and practice matched controls.
Mean age of participants	NA	NA	median age of 53 years old.	56.6 years old	cases: 60.2 years old; controls: 59.7	55.9 years old	NA
Percentage of females	Study I: 31.6%, Study II: 34.6%	88.30%	34.30%	35.50%	100%	NA	NA
Diagnosis of Chronic Liver Disease	Clinical records from the Taiwan’s National Health Insurance Programme between 2000-2003 and 2004-2013 were used in addition to ICD-9-CM codes to identify patients’ medical conditions and complications with Chronic Liver Disease.	The General Practice Research Database (GPRD) were used to extract the records of all persons between June 1987 and April 2002 with a recorded diagnosis of PBC using OXMIS an READ code.	Based on ICD 10 codes idetyfing patient with a diagnosis of cirrhosis with and without hepatic encephalopathy.	Patients were included if they had been discharged with one of the International Classification of Diseases, 10th edition codes. Diagnosis of alcoholic fibrosis and sclerosis of liver, alcoholic cirrhosis, primary biliary cirrhosis, secondary biliary cirrhosis, biliary cirrhosis, unspecified, autoimmune hepatitis, and other specified inflammatory liver disease were included. Viral cirrhosis was not included in this analysis.	Case notes for the patients were reviewed. For this study, a diagnosis of PBC was made if two of the following three criteria were met: abnormal liver function tests with a cholestatic pattern, a liver biopsy report consistent with PBC or positive antimitochondrial antibodies.	PBC used as an exposure factor.	ICD 10 code for cirrhosis, esophageal varices and portal hypertension.
Diagnosis of Fractures	Clinical records from the Taiwan’s National Health Insurance Programme between 2000-2003 and 2004-2013. ICD-9-CM codes were used to identify patients’ medical conditions with different typyes of fracture and injury included skull, neck, trunk, upper and lower limb fractures.	The General Practice Research Database (GPRD) were used to extract the records of all persons between June 1987 and April 2002 with any incident hip and radius/ulna fractures. In addition to potential confounders: height, weight, and smoking habit, drug exposures (eg, oral and injected corticosteroids) and ursodeoxycholic acid use.	Based on ICD 10 codes identyfing patients with a diagnosis of fracture in the skull, back, lower or upper limbs.	Fractures were identified using the International Classification of Diseases, 10th edition codes. Fractures of the skull and facial bones, cervical spine, thoracic spine, ribs, pelvis, lumbar spine, shoulder, humerus, upper forearm, lower forearm, wrist and hand, proximal femur, lower femur, lower leg, ankle, foot, and,finally, osteoporotic fracture were included. Fractures of the spine, humerus, distal forearm, and proximal femur were considered as low-trauma osteoporotic fractures.	All PBC patients and controls were sent a 9-page questionnaire enquiring about their fracture experience.	Osteoporosis or a fracture as an outcome	ICD code for hip and wrist fractures
Adjusted OR/HR/IRR	Study I: HR 1.83, 95% CI 1.67 to 2.01, Study II: higher risks for post-fracture sepsis (OR 1.77, 95% CI 1.60 to 1.96), acute renal failure (OR 1.63, 95% CI 1.33 to 1.99), and 30-day in-hospital mortality (OR 1.61, 95% CI 1.37 to 1.89) were associated with previous LC	Risk of any fracture: (HR: 2.04; 95% CI: 1.70 –2.44) hip fracture: (HR: 2.14 (95% CI: 1.40 –3.28) ulna/radius fracture: (HR: 1.95 (95% CI: 1.42–2.69)	IRR 1.63 (95% CI 1.69–2.05, p = 0.001)	The adjusted hazard ratio (HR) for any fracture was 2.4 in patients with cirrhosis (95% confidence interval [CI], 2.2–2.5)	No statistically significant increases in their risk of ‘low impact’ fracture compared with the general population (OR: 1.0, 95% CI: 0.31–2.68). Although the overall bone fracture risk was moderately increased (OR: 1.5, 95% CI: 0.8–2.89) this finding was not statistically significant.	PBC patients had an OR of 1.86 (95%CI 1.54 to 2.24, P < 0.00001)	Adjusted HR for fracture: compensated cirrhosis 4.1 (95% CI 3.0 to 5.40)
Confounder adjustment	Multiple cox proportional hazard and multiple logistic regression models to control the confounding effects of medical conditions when investigating the risks and outcomes of fracture in patients with LC in studies I and II were used.	Cox regression to estimate the hazard ratios for any fracture, hip fracture, and ulna/radius fracture in the PBC cohort compared with the general population.	Each HE patient was matched with one cirrhotic patient without HE and one non-cirrhotic patient (1:1:1) by age, sex, and comorbidities at the same enrollment date [17]. The same exclusion criteria were applied to both matched cohorts.	Univariate and multivariate cox proportional hazard models to assess the hazard ratio (HR) with 95% confidence interval (CI). Persons with missing data were excluded from the analyses.	The cases and controls were closely matched for common risk factors for osteoporosis including age, age of menarche and menopause, use of hormone replacement therapy (HRT).	The different studies were matched for age and gender.	They were matched for age, gender.
Quality assessment (Newcastle-Otawwa scale)	Selection: 4 Comparability: 1 Outcome: 3	Selection: 4 Comparability: 1 Outcome: 3	Selection: 3 Comparability: 1 Outcome: 3	Selection: 4 Comparability: 1 Outcome: 3	Selection: 2 Comparability: 1 Outcome: 1	Selection: 3 Comparability: 1 Outcome: 3	Selection: 3 Comparability: 1 Outcome: 2

All investigators performed the data extraction process independently to ensure accuracy. Any discrepancy in data was resolved by referring back to the original articles.

Statistical analysis

Data analysis was performed using Review Manager 5.3 software from the Cochrane Collaboration (London, United Kingdom). Adjusted point estimates and standard errors from the individual studies were combined using the generic inverse variance method of DerSimonian and Laird, which assigned the weight of each study based on its variance [16]. In light of the possible high between-study variance due to different study designs and populations, we used a random-effect model rather than a fixed-effect model [15]. Cochran's Q test and I2 statistic were used to determine the between-study heterogeneity. A value of I2 of 0%-25% represents insignificant heterogeneity, greater than 25% but less than or equal to 50% represents low heterogeneity, greater than 50% but less than or equal to 75% represents moderate heterogeneity, and greater than 75% represents high heterogeneity [14,15].

## Results

**Figure 2:**
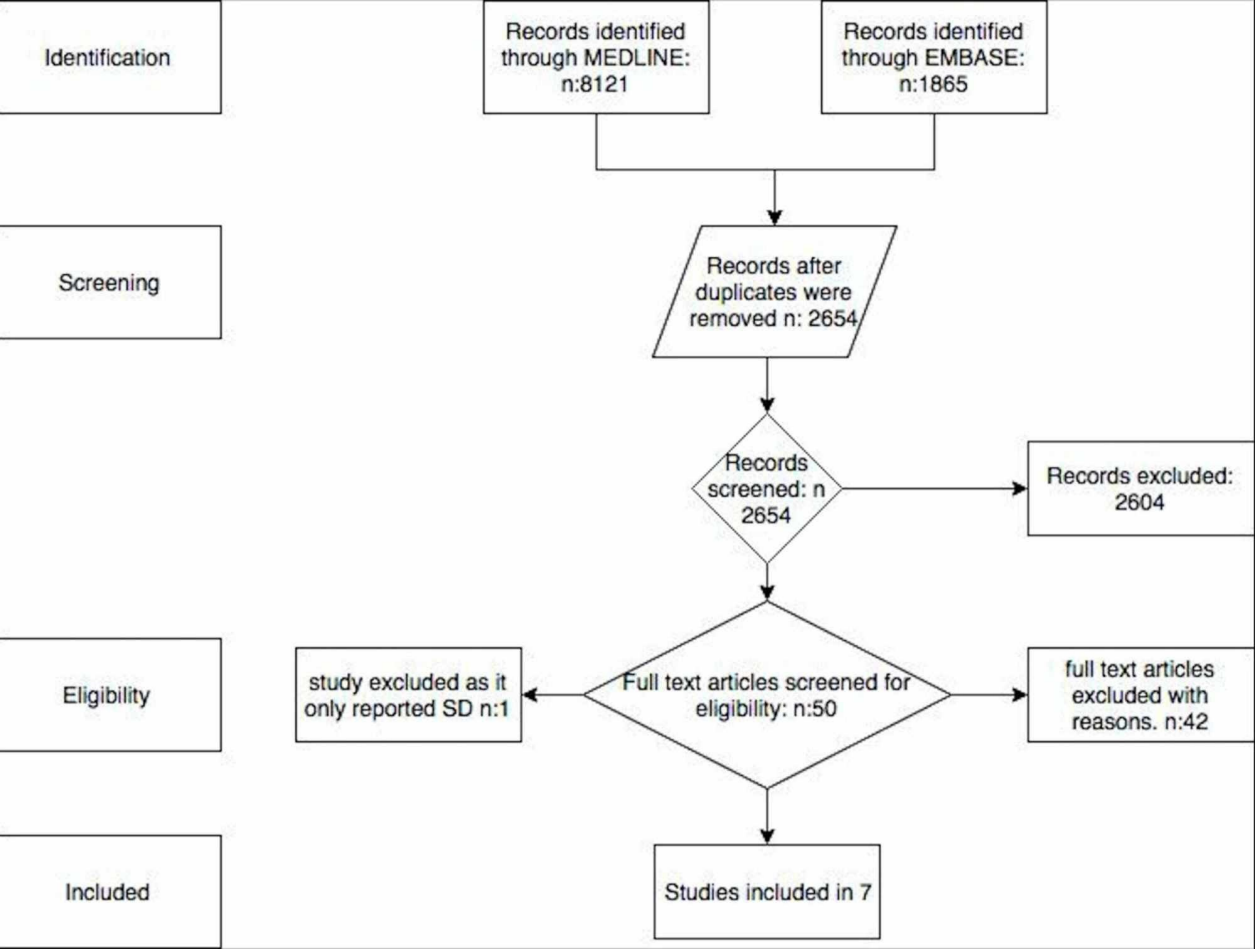
Search criteria and eligibility

A total of 9986 articles were obtained. After excluding duplicates, a total of 2645 articles underwent title and abstract review. A total of 2604 articles were excluded, as they were case reports, book articles, letters to the editor, or review articles without the information needed for the analysis, leaving 50 for a full-length article review. A total of 42 articles were dismissed at this time because they did not have any comparators. An extra article was dismissed since it only included SD. A total of seven studies were used for statistical analysis; those studies were cohort, cross-sectional, and case-report studies. Those studies fulfilled the eligibility criteria established during the analysis. The outlines of the literature review and study selection process are given in Figure 2. The clinical characteristics of each study and the quality assessment are described in Table 1.

**Figure 3:**
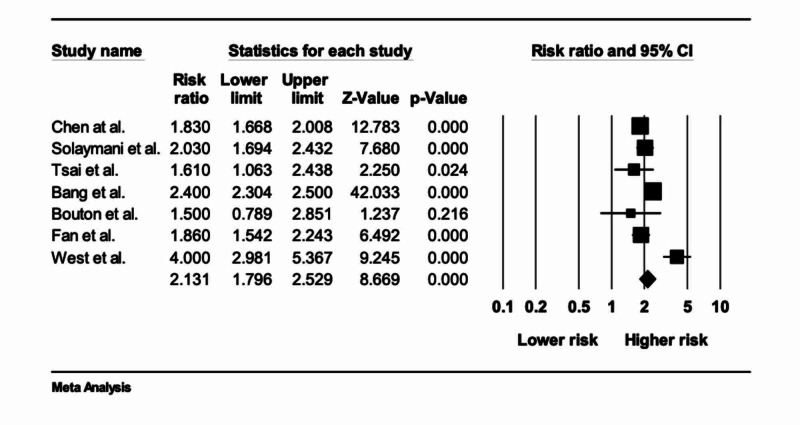
Relative risk and P-value

This study found an increased risk of osteoporotic fractures in patients with CLD vs patients who did not have CLD. Pooled odds ratio (OR) of 2.13 (95% CI, 1.79 - 2.52), p<0.001, as shown in Figure 3.

The Cochran’s Q test and I2 were calculated to measure the heterogeneity among the studies. The I2 calculated for this study was 88.5, representing high heterogeneity among the studies. The Egger regression test and funnel plots were used to assess publication bias. Egger's regression test (p=0.44) did not show a publication bias.

## Discussion

This meta-analysis study was performed by the research team to assess the risk to develop osteoporotic fractures in patients diagnosed with chronic liver disease. After evaluating the seven studies that met the inclusion criteria, the calculated odds of developing an osteoporotic fracture in patients with CLD was found to be 2.13 times the odds of the controls who did not have CLD. 

Our meta-analysis includes studies from around the world, such as Taiwan, United Kingdom, Denmark, and China with the involvement of more than 990,000 participants and their medical records [18-24]. Chen et al. reported that liver cirrhosis (LC) is an important risk factor for fracture with the adjusted HR of fracture being 1.83 (95% CI 1.67 to 2.01), with more medical complications and 30-day in-hospital mortality after fracture [18]. Similarly, Solaymani et al. demonstrated that people with primary biliary cirrhosis (PBC) are approximately at a two-fold increased risk for any fracture in comparison with the general population [19]. Both of these studies were consistent with our findings (OR of 2.13; 95% CI 1.79 - 2.52) [18,19]. These results validate an association of CLD with osteoporotic fractures. In addition, all of our included studies had a satisfactory selection criteria and outcomes except the study done by Boulton et al., which had 100% females and therefore is an under-representation of the general population. Although it was the first study that had revealed the risk of osteoporosis in PBC patients, the sample size was not large enough and it was a self-reported questionnaire-based study. Because of this reason we scored it low on the outcomes. 

Although the liver is involved in multiple metabolic pathways, the exact mechanism by which CLD can lead to osteoporotic fractures is not well understood. Liver disease leads to decreased formation of 25-OH-vitamin D which causes impaired bone resorption, bone mineralization, and decreased calcium resorption in the gastrointestinal tract [7]. CLD causes increased concentrations of certain cytokines like interleukin (IL)-1, IL-6, and tumor necrosis factor α (TNFα), thereby increasing the osteoclastic activity by stimulating the production of receptor activator of nuclear factor kB ligand (RANKL) [25,26]. Also, CLD can cause vitamin K deficiency which is an important vitamin for the synthesis of an osteoblast-specific protein, osteocalcin. Unconjugated bilirubin excess in the liver disease can also interfere with the activation of the primary osteoblasts to perform their function [26,27]. These are some of the mechanisms which can potentially cause osteoporosis in chronic liver disease leading to fractures [28].

A deeper understanding of the mechanisms described above could help the medical community to develop prophylactic and preventive measures. Finding different ways to modify risk factors and behaviors can decrease the negative effect that they have on healthy bone metabolism. By these means, we can improve the morbidity associated with osteoporotic fractures in patients with chronic liver disease.

Regarding the strengths of the study, it includes research studies done around the world with inclusion of over 990,000 patients. There was no publication bias found using the Egger regression test. Our study does have some limitations such as it included only observational studies. Also, only seven studies were eligible, out of which some were medical registry-based. The results showed high heterogeneity among these studies but there was no publication bias. We could not include a study by Patel 2009, as it reported only OR and SD. The result from Patel had very low SD, but unfortunately they did not report standard error.

More prospective studies are needed to better understand the mechanism of the risk factors in order to let us work more on preventive measures to decrease the morbidity associated with the fractures. Early detection and lifestyle modification could potentially decrease the risk of osteoporosis and hence the fractures in patients with CLD. This work has been presented as an abstract [29].

## Conclusions

We found a significant association between CLD and the risk of developing osteoporotic fractures. The calculated risk was 2.13 times higher for patients with CLD when compared with controls. The results showed high heterogeneity but no publication bias. More prospective studies are needed to fully understand the mechanisms involved in loss of bone density and osteoporotic fractures in order to improve the morbidity associated with this disease. 
